# Assessing healthcare efficiency and determinants in China: the perspective of provincial-level social health expenditure

**DOI:** 10.1186/s12913-025-12767-w

**Published:** 2025-04-22

**Authors:** Chuanhai Xu, Jieyao Chen, Yuehui Li, Dongyu Huang, Yuan Xue

**Affiliations:** 1 Department of Operational Management, Provincial Hospital Affiliated to Fuzhou University, Dong Jie Street, Fuzhou, Fujian 350001 China; 2 Department of Operational Management, Fujian Provincial Hospital, Fuzhou, Fujian 350001 China; 3 School of Basic Medical Science, Fujian Medical University, Fuzhou, Fujian 350001 China

**Keywords:** Social health expenditure, Efficiency, Malmquist, DEA

## Abstract

**Objective:**

Social healthcare expenditure is a crucial component of global healthcare expenditure. Assessing the relative efficiency, dynamic changes, and influencing factors of province-level social investment in China would facilitate the continued and stable development of healthcare services.

**Methods:**

Based on the Malmquist Data Envelopment Analysis and Tobit regression model, this study pursued panel data from 2012 to 2020 for a set of 31 provinces and cities in China to estimate the efficiency of social health expenditure and explore the factors that influence the efficiency.

**Results:**

All provinces experience a decline in the total factor productivity change index in the period 2012–2020. The average value is 0.917, which implies a deterioration of 8.3% per year. Most provinces show increased efficiency change but experience declines in technical progress, except Sichuan, Guangdong, and Shandong. Although efficiency change shows an upward trend, the positive impact is offset by technological regression, leading to a decline in overall productivity. Population density and urbanization level are found to be associated with expenditure efficiency in opposite directions. Higher levels of urbanization are associated with higher efficiency scores and the opposite impact is observed for population density.

**Conclusions:**

This study shows that there is still much room for improving the efficiency of province-level social health expenditure and reinforcing the imperatives to increase investment in technological progress in health to foster the efficiency of social healthcare expenditure. Policy reforms and adjustments tailored to the specific conditions of different regions may be necessary for better alignment.

## Introduction

The goal of achieving universal health coverage worldwide has become a major objective of the World Health Organization, and access to high-quality care is a priority worldwide [1]. As China experiences socio-economic development, population aging, the prevalence of non-communicable chronic diseases, and heightened health awareness, the demand for healthcare services has surged, which has resulted in rising healthcare costs. Despite the sustained increase in the government’s investment in healthcare, the supply of healthcare services has not kept pace with the growing demand. This has resulted in an imbalance between supply and demand in the healthcare system, conflicts in doctor-patient relationships, and rapidly escalating healthcare spending. China’s total health expenditures as a share of gross domestic product (GDP) have risen from 4.55% in 2008 to nearly 7.12% in 2020, outpacing GDP growth (6.1% in 2019; shocked by the pandemic of COVID- 2019, GDP growth in 2020 was 2.3%). However, in a context of constrained government fiscal space and the long-term unsustainability of continuously increasing health investments, it is challenging to address rising healthcare costs and growing demand for healthcare services solely through government health expenditures.

Recent data shows that in 2022, China’s total health expenditure amounted to 8,484.67 billion yuan, with government health expenditure accounting for just 28.2% (2,391.64 billion yuan). In contrast, social health expenditure reached 3,801.58 billion yuan, with a proportion of 44.8%. While existing studies have advanced understanding of healthcare input efficiency [2–4], some scholars have noted that the societal dimension of healthcare efficiency remains limited [5–9]. Current research focuses mainly on assessing the efficiency of provincial government health expenditure [10–13]. Yet, given that the government health expenditure comprises only 28% of total health expenditure, while social health expenditure accounts for a much larger share at 44.8% in 2022. Consequently, a one-dimensional estimation of the efficiency of government health expenditures fails to capture the full picture of healthcare efficiency in China. This disconnect may explain why, despite the Chinese government’s repeated emphasis on the accelerated growth of government health expenditures, the public still perceives that it is expensive to seek medical services.

Despite the issue’s importance, to the best of our knowledge, there are still not many studies that have examined the efficiency of social health inputs in China, and the exploration of the factors influencing this efficiency is virtually nonexistent. Considering the critical role of social health inputs in the sustainable development of healthcare, understanding and grasping their efficiency and trends, as well as identifying potential influencing factors, are crucial. To fill this gap, therefore, the present study is conducted to explore and analyze the efficiency and determinants of social health expenditures at the provincial level in China from 2012 to 2020, utilizing the DEA model, the Malmquist index model, and the Tobit model.

## Materials and methods

### Data sources

The data utilized in this study was derived from the *China Statistical Yearbook*, *Local statistical yearbooks*, and *the China Health Statistical Yearbook*. To ensure data availability and continuity, the present study selected statistical data spanning the years from 2012 to 2020, covering 31 provinces in mainland China with 279 observations. We take 31 provinces as decision-making units (DMUs). Drawing lessons from Li et al. [12, 14], we employ the social health expenditure in each province and per capita health costs as the input variables. Output variables are selected to avoid fuzzy health indicators, such as life expectancy, infant mortality rate, and so on [15], but rather include the number of medical and health institutions [16, 17], the number of beds in medical institutions [16] and the number of health care technicians [18], representing the delivery capacity of healthcare services. To explore the effect of the demographic, economic, education, and urbanization degree on the efficiency, per capita GDP, population density, the illiterate population aged 15 and over, and the proportion of urban population at year-end are included in the Tobit regression analysis. Table 1 displays an overview of all the variables used in the present study.

**Table 1:** Selection of input, output, and relationship variables

Model	Type	Indicators
DEA and Malmquist Model	Inputs	Social health expenditure
		Per capita health costs
	Outputs	Medical and health institutions
		Bed of medical institutions
		Health technicians
Tobit Model	Economic	Per capital GDP
	Education	Illiterate Population Aged 15 and Over
	Urbanization	Proportion of Urban Population at Year-end
	Demography	Population density

## Methods

### DEA model

The DEA model has been widely applied to the assessment of efficiency in various fields. Given the complexity of the healthcare system, the DEA model is considered to be one of the most important methods for evaluating the efficiency of healthcare services. The DEA model is a non-parametric technique that constructs an effective convex surface of the production frontier through linear programming. This frontier surface is formed by a series of effective DMUs connected by linear line segments, and the relative efficiency of each DMU is derived based on the relative distance of inefficient observations from the frontier, which can effectively deal with the situation of multiple inputs and multiple outputs without the need to predetermine the specific form of the production function. According to the assumption of returns to scale, the DEA model includes the CCR-DEA model and the BCC-DEA model, which correspond to the assumption of constant returns to scale (CRS) and variable returns to scale (VRS), respectively. We use an input-orientation VRS model to compute efficient scores through the following linear programming problems:

1 $$\begin{gathered} {\hbox{max} _{\varphi,\lambda }}\varphi \hfill \\ \varphi {\text{ s}}{\text{.t}}{\text{. }} - \varphi {y_i}+Y\lambda \geq 0 \hfill \\ {x_i} - X\lambda \geq 0 \hfill \\ I{1^\prime }\lambda =1 \hfill \\ \lambda \geq 0 \hfill \\ \end{gathered}$$

$\varphi$ is a scalar (satisfying 1 ≤ $\:\phi\:$ ≤ + $\infty$), and 1/$\varphi$ defines the technical output efficiency score, varying between zero and one.

### Malmquist-DEA model

The DEA model measures the relative efficiency of each DMU at a certain point in time, and cannot measure the dynamic change of the efficiency, while the Malmquist-DEA could address the above issue. The Malmquist index can evaluate DMU’s efficiency by year, and the simultaneous application of the DEA and the Malmquist mode not only can statically measure the efficiency score of a certain year but also monitor the change of the efficiency score in a certain consecutive time, which can be more comprehensive to evaluate the efficiency of DMUs. The *Total Factor Productivity Change* (TFPCH) could be decomposed into favorable *Technical Progress* (TECH) and *Technical Efficiency Progress Change* (EFFCH), and the *Technical Efficiency Change* could be further divided into *Pure Efficiency Change* (PECH) and *Scale Efficiency Change* (SECH). This approach has proven itself to be a good tool for measuring the *TFPCH* growth of DMUs [19].

2 $$TFPCH = TECH \times EFFCH = TECH \times PECH \times SECH$$

The values of *TPFCH*, *EFFCH*, *TECH*, *PECH*, and *SECH* above one mean improvement, while below one indicates a decline.

### Tobit model

In this study, the overall efficiency of social health expenditure inputs and outputs calculated by the BCC-DEA model was used as the dependent variable to further explore the effect of exogenous factors on efficiency. Since the overall efficiency produced by the DEA model lies between zero and one, which is truncated and censored data, the use of the traditional ordinary least squares method may lead to biased estimation. The Tobit model also called a censored regression model, describes the relationship between the left or right censored continuous dependent variable and independent variables by the maximum likelihood method, which was originally proposed by James Tobin [20]. The model form can be written as:

3 $$\begin{gathered} y_{{it}}^{*}={x_{it}}^{\prime }\beta +{\gamma _{it}}+{v_{it}} \hfill \\ {y_{it}}=\left\{ {\begin{array}{*{20}{l}} 0&{{y_{i{t^*}}} \leq 0} \\ {{y_{i{t^*}}}}&{0<{y_{it}}^{*}<1} \\ 1&{y_{{it}}^{*} \geq 1} \end{array}} \right. \hfill \\ \end{gathered}$$

Where, $\:{y}_{it\:}^{*}$is the overall efficiency value that is calculated from the DEA model in each province from 2012 to 2020. *x* refers to a group of explanatory variables, including per capita GDP, urbanization rate, population density, and education level. $\:\gamma\:$ is the unobserved regional effect. $\:v$ is the random disturbance term. Subscript $\:i$ and $\:t$ represent $\:i$ province and $\:t$ times. Since the Tobit model is non-linear, using fixed-effects in panel Tobit analysis would cause incidental parameter problems as *N* increase, leading to biased estimates [21]. Moreover, beyond the incidental parameters issue, fixed-effects Tobit model suffer from complications related to the distribution of disturbance variance estimator [22]. The random-effects Tobit model offers a more efficient estimation framework by effectively capturing both cross-sectional and temporal variations while addressing the biases inherent in the fixed-effects approach [10]. Therefore, we employed the panel Tobit model with random effects to ensure a more efficient and reliable estimation.

### Statistical analysis

This study is conducted in three steps. First, descriptive statistics is conducted using the software SPSS, version 23. Second, the DEAP 2.1 software is used to compute the overall efficiency of each province from 2012 to 2020, the total factor productivity index, and the geometric mean of its decomposition components. Third, the random-effects panel Tobit model is performed in Stata 15 to explore potential factors that influence the overall efficiency. We used two-sided significance tests for all the analyses, with statistical significance set at *p* < 0.05.

## Results

### Descriptive statistics

Table 2 shows changes in input and output variables from 2012 to 2020. The social health expenditure of the provinces increased annually. Health investment in 2020 exceeded 3 trillion for the first time, which was approximately three times that in 2012. The per capita health costs increased by 2.5-fold from 2012 to 2020. In terms of output, the numbers of medical institutions, beds, and health technicians had increased by varying margins.

**Table 2:** Descriptive statistics of inputs and outputs from 2012 to 2020

Year	Inputs		Outputs		
	Social Health Expenditure (billions,¥)	Per capita health costs (yuan,¥)	Number of Medical and Health Institutions	Numbers of Bed	Numbers of Health Technicians
2012	10030.70	2068.8	950,297	5,724,775	9,115,705
2013	11393.79	2316.2	974,398	6,181,891	9,790,483
2014	13437.75	2565.5	981,432	6,601,214	10,234,213
2015	16506.71	2962.2	983,528	7,015,214	10,693,881
2016	19096.68	3328.6	983,394	7,410,453	11,172,945
2017	22258.81	3756.7	986,649	7,940,252	11,748,972
2018	25810.78	4206.7	997,433	8,404,088	12,300,325
2019	29150.57	4669.3	1,007,579	8,806,956	12,928,335
2020	30273.67	5111.1	1,022,922	9,100,700	13,474,992

### Analysis of dynamic changes in efficiency of provincial-level social health expenditures in China

**Fig. 1:**
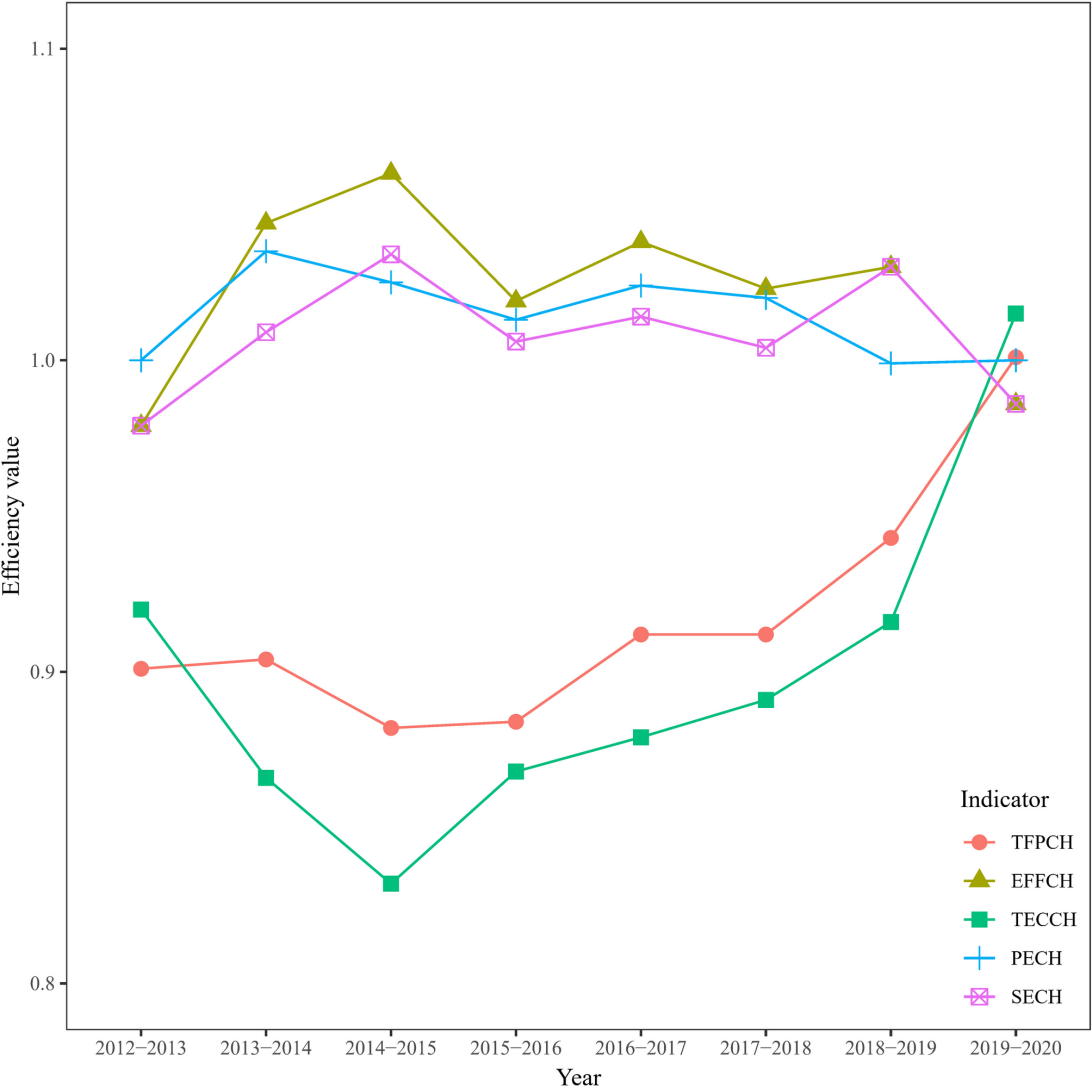
Trends of social health expenditure efficiency from 2012 to 2020

As shown in Table 3, the efficiency of social investment is relatively stable and has increased overall. The indicators *EFFCH*, *TECH*, *PECH*, and *SECH* reflect the efficiency of societal inputs as well as technological processes in healthcare. In terms of *TFPCH*, the indicator peaked at 1.001 in 2019–2020, signaling growth. Whereas the indicator mostly hovered at 0.9 during previous years, particularly in 2014–2015 and 2015–2016, declining to its lowest values of 0.882 and 0.884. The average value of 0.917 implies a decrease of 8.3% per year. The *EFFCH* indicator is above 1 for the majority of the period from 2012 to 2020, indicating an improvement in technology efficiency. But it declines by 2.1% and 1.4% during 2012–2013 and 2019–2020, respectively, showing some volatility. The mean value of *EFFCH* was 1.022, which represents an average annual growth rate of 2.2%. The *TECH* indicator reaches a maximum value of 1.015 in 2019–2020, while in the remaining years that are less than 1. The average value of 0.897 for this indicator indicates technological regression during the period from 2012 to 2020. For the *PECH* indicator, the minimum value is close to 1 (0.999), which grows at a steady pace. Similarly to *PECH*, changes in scale efficiency were generally on an upward trend, but it is worth noting that a decline is observed in 2019–2020. As shown in Fig. 1, the change of the *TFPCH* is essentially identical to that of the *TECH*. Although the *EFFCH* shows an increasing trend, the *TFPCH* indicator is generally less than 1 due to the effect of the drop in the *TECH*.

**Table 3:** Malmquist index and their decomposition of social healthcare inputs and outputs, 2012–2020

Year	TFPCH	EFFCH	TECH	PECH	SECH
2012–2013	0.901	0.979	0.920	1.000	0.979
2013–2014	0.904	1.044	0.866	1.035	1.009
2014–2015	0.882	1.060	0.832	1.025	1.034
2015–2016	0.884	1.019	0.868	1.013	1.006
2016–2017	0.912	1.038	0.879	1.024	1.014
2017–2018	0.912	1.023	0.891	1.02	1.004
2018–2019	0.943	1.030	0.916	0.999	1.030
2019–2020	1.001	0.986	1.015	1.000	0.986
Mean	0.917	1.022	0.897	1.014	1.008

### Analysis of region efficiency of provincial-level social health expenditures in China

**Fig. 2:**
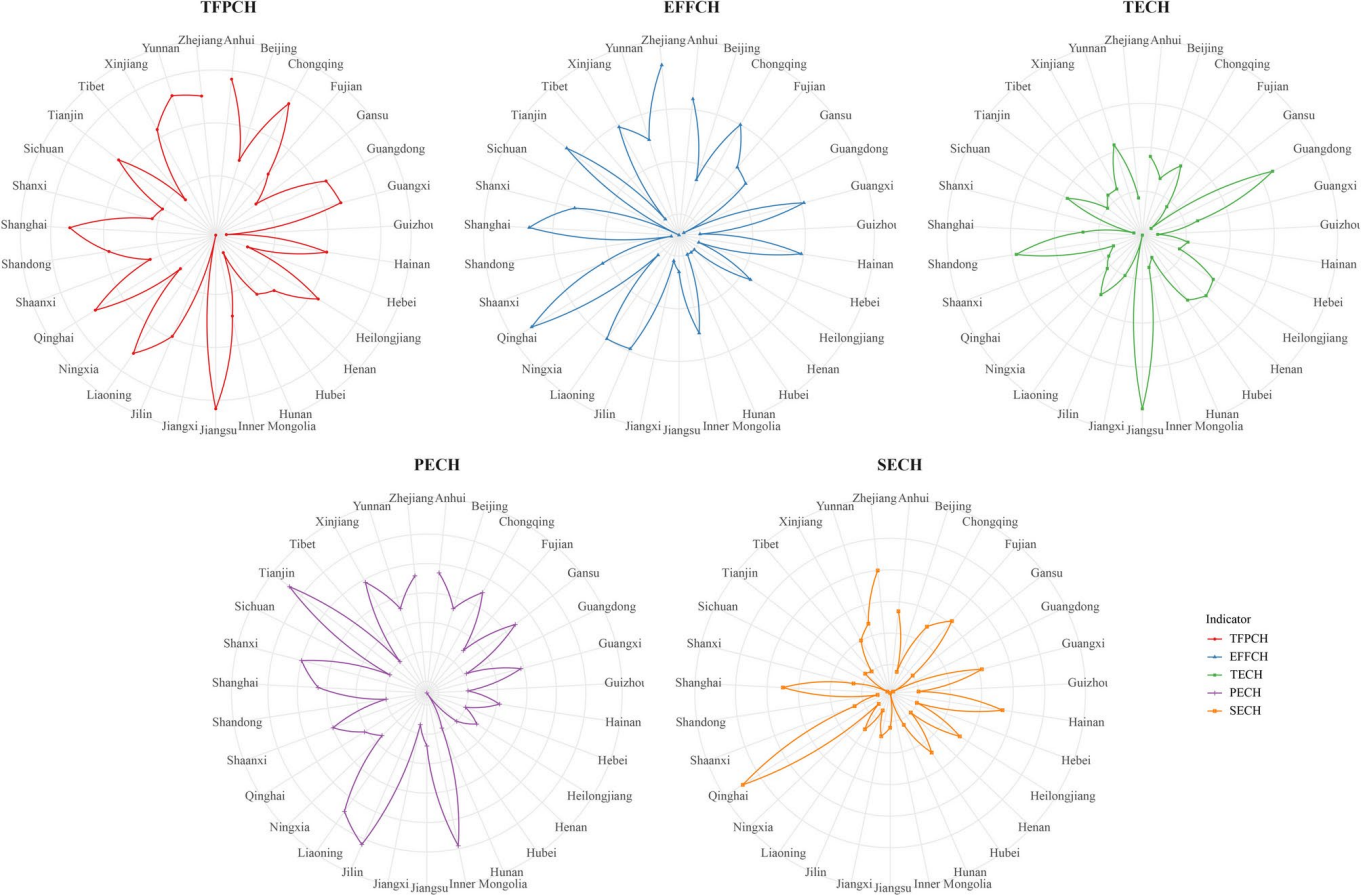
Trends of social health expenditure efficiency in 31 provinces from 2012 to 2020

According to Table 4, there are considerable differences in the performance of social health expenditures between different provinces in China. Qinghai shows the greatest performance improvement in the indicator *EFFCH* at 1.058. while Sichuan is the lowest at 0.992, showing a decreasing trend. For the *TECH* indicator, the largest figure was Jiangsu (0.949), and other provinces in the country show a declining trend in this indicator, with approximately half of the provinces below 0.9. Tianjin shows the greatest improvement in the *PECH* indicator, and conversely, Hubei is decreasing at the greatest rate, suggesting that more resources should be invested in staff training and equipment upgrading in Hubei. More than three-quarters of the provinces displayed an increasing trend in the *SECH* indicator, most notably Qinghai at 1.046, while Inner Mongolia had the worst performance at 0.991. The *TFPCH* indicator was less than 1 in all provinces, with Jiangsu, Anhui, and Chongqing in the top three, and Hunan, Guizhou, and Jiangxi in the bottom three. About a quarter of the provinces had a *TFPCH* of less than 0.9, implying an average annual decline of more than 10%. Figure 2 displays the distribution of the 31 provinces on the 5 indicators by radar chart.

**Table 4:** Malmquist index and its decomposition of social health expenditures by Province

Province	TFPCH	EFFCH	TECH	PECH	SECH
Anhui	0.946	1.044	0.906	1.027	1.017
Beijing	0.909	1.014	0.897	1.016	0.998
Fujian	0.910	1.026	0.887	1.005	1.021
Gansu	0.896	1.024	0.875	1.024	1.000
Guangdong	0.930	0.994	0.936	1.001	0.992
Guangxi	0.933	1.041	0.896	1.019	1.021
Guizhou	0.877	1.000	0.877	1.000	1.000
Hainan	0.925	1.039	0.891	1.011	1.027
Hebei	0.888	1.000	0.888	1.000	1.000
Henan	0.910	1.000	0.910	1.000	1.000
Heilongjiang	0.929	1.024	0.908	1.006	1.017
Hubei	0.906	1.000	0.906	0.986	1.014
Hunan	0.881	1.000	0.881	0.999	1.002
Jilin	0.924	1.039	0.890	1.042	0.997
Jiangsu	0.954	1.006	0.949	1.004	1.002
Jiangxi	0.872	1.002	0.870	0.997	1.005
Liaoning	0.940	1.040	0.903	1.035	1.005
Inner Mongolia	0.911	1.030	0.885	1.039	0.991
Ningxia	0.895	1.003	0.892	1.007	0.996
Qinghai	0.939	1.058	0.888	1.011	1.046
Shandong	0.923	0.995	0.928	1.000	0.995
Shanxi	0.903	1.033	0.874	1.030	1.003
Shaanxi	0.905	1.023	0.884	1.020	1.003
Shanghai	0.941	1.049	0.897	1.023	1.025
Sichuan	0.900	0.992	0.908	1.000	0.992
Tianjin	0.930	1.046	0.890	1.045	1.001
Tibet	0.894	1.000	0.894	1.000	1.000
Xinjiang	0.929	1.039	0.894	1.029	1.010
Yunnan	0.941	1.030	0.913	1.016	1.014
Zhejiang	0.938	1.057	0.887	1.026	1.030
Chongqing	0.943	1.040	0.906	1.025	1.015

### Analysis of factors influencing the efficiency of provincial-level social health expenditures in China

To explore the effect of demographic, economic, urbanization, and education on the efficiency of province-level social health expenditure. We conducted a random-effects panel Tobit model to identify the influencing factors. Table 5 reports the estimated result of the Tobit model. Urbanization rate and population density are found to have a significant effect on the overall efficiency (*p* < 0.05). Specifically, population density has a negative impact on efficiency. As population density increases, a degradation in efficiency is observed. Whereas the urbanization level has a statistically significant positive effect on the efficiency. The efficiency increases when the urbanization degree goes up. Per capita GDP and education level were not detected to contribute meaningfully to efficiency.

**Table 5:** The estimated results of the Tobit model analysis

Relative variables	β	standard error	t-value	*p*-value	[95% Confidence Interval]	
					Lower	Upper
Per capita GDP	1.75E- 07	4.35E- 07	0.40	0.687	− 6.77E- 07	1.03E- 06
Education level	1.23E- 08	1.25E- 08	0.99	0.324	− 1.22E- 08	3.68E- 08
Urbanization rate	0.0126309	0.0016112	7.84	0.00	0.009473	0.0157889^***^
Population density	− 0.00033	0.0000812	− 4.06	0.00	− 0.0004892	− 0.0001708^***^
Constant	0.1282082	0.1222538	1.05	0.294	− 0.1114047	0.3678212
sigma_u	0.3088932	0.0466937	6.62	0.000	0.2173752	0.4004113^***^
sigma_e	0.0604163	0.0031149	19.4	0.000	0.0543111	0.0665214^***^

***p<0.01, **p<0.05, *p<0.1

### Robustness test

To evaluate the stability of the significant factors identified in the previous model across different sample compositions, we excluded Tibet and Xinjiang from the sample and re-estimated the model. As shown in Table 6, the core determinants of urbanization rate and population density remain statistically significant (*p* < 0.01), with only marginal fluctuations in their estimated coefficients. Additionally, the remaining explanatory variables also do not reach statistical significance, further confirming the stability of the findings obtained from the original model.

**Table 6:** The estimated results of the robustness test

Relative variables	β	standard error	t-value	*p*-value	[95% Confidence Interval]	
					Lower	Upper
Per capita GDP	2.47E- 07	4.25E- 07	0.58	0.561	− 5.86E- 07	1.08E- 06
Education level	1.09E- 08	1.22E- 08	0.89	0.371	− 1.30E- 08	3.48E- 08
Urbanization rate	0.0117442	0.0016156	7.27	0.000	0.0085777	0.0149107^***^
Population density	− 0.0003241	0.0000817	− 3.97	0.000	− 0.0004842	− 0.0001639^***^
Constant	0.1824071	0.1200836	1.52	0.129	− 0.0529525	0.4177666
sigma_u	0.3091137	0.0477047	6.48	0.000	0.2156141	0.4026133^***^
sigma_e	0.0588427	0.0031121	18.91	0.000	0.052743	0.0649424^***^

***p<0.01, **p<0.05, *p<0.1

## Discussion

The present study analyzes the efficiency and change of province-level social health expenditures in China from 2012 to 2020 and identifies factors that affect the performance of these inputs and outputs. The findings reveal a substantial opportunity for improving efficiency, with a decline in total factor productivity primarily attributed to technological regression, leading to reduced output with the same input. Population density and urbanization degree may emerge as significant factors affecting efficiency.

The dynamic efficiency of expenditure, as estimated by the Malmquist-DEA model, shows an average *TFPCH* of 0.917 for provincial social health expenditure from 2012 to 2020, indicating an overall downward trend. This is consistent with the findings of Li et al. [14]. As far as the *TFPCH* decomposition indicator is considered, the average value of *TECH* is 0.897, signifying a decline in technological progress. Conversely, the efficiency change (*EFFCH*) has an average value of 1.022, with its components, pure efficiency change (*PECH*) and scale efficiency change (*SECH*), at 1.014 and 1.008, respectively, indicating an upward trend. The two components of *TFPCH* move in opposite directions, which ultimately dilutes the overall growth of total factor productivity by counteracting each other’s effects, and the 2.2% increase in *EFFCH* is offset by technological regression. The annual decline in the growth rate of technological change is the main reason for the drop in total factor productivity, a finding supported by Al-Hanawi and Makuta et al., who observed a 5.6% annual decrease in healthcare *TFPCH* mainly due to technological regression [23]. Similarly, other studies also reported that the observed drop in productivity was mainly attributed to technological degradation rather than efficiency changes [24]. All provinces exhibit a *TECH* value of less than 1, which indicates that they have all experienced technological regression. All provinces have shifted closer to the origin on the production frontiers rather than away from it. Notably, Guangdong, Shandong, and Sichuan also have an *EFFCH* value of less than 1, making them the only three provinces experiencing declines in both efficiency changes and production frontiers. While investment in technology may increase healthcare costs, it is also a key driver of productivity gains [25]. Addressing existing technological barriers by investing in the R&D of new technologies, introducing advanced medical technologies and management concepts, and strengthening staff training could unlock potential benefits for productivity in healthcare [23, 26].

Regarding factors influencing the efficiency of health expenditure, this study finds that population density and urbanization rate are both associated with the efficiency of social health expenditures. Population density shows a negative relationship with efficiency, consistent with the findings by Athanassopoulos et al. [27], but contrary to some scholars’ findings higher population density would reduce the costs of management and supervision, resulting in the problem of economies of scale, and thus enhancing expenditure efficiency [28]. In a densely populated country like China, the problem of inadequate and overly expensive medical services is a persistent challenge that China’s health care system is facing. In regions with high population density, limited health expenditures are required to cover a larger population, and similarly, scarce healthcare resources have to be used to fulfill the growing healthcare service demand, imposing pressure on the allocation of resources and affecting expenditures efficiency to a certain extent. Moreover, multiple environmental factors, such as food waste, greenhouse gas emissions, and temperature changes, which accompany high-density populations contribute to a sustained increase in healthcare demand, further exacerbate the burden on the region’s health systems and thus reducing the efficiency of healthcare expenditure [29, 30]. Accelerated urbanization and rapid development of public transport have led to a redistribution of population, increasing population density in some areas. However, public policies and social welfare initiatives that rely on household registration rather than the actual region of residence for their design and implementation in China leave the mobile population with insufficient or restricted access to many social welfare benefits [31, 32], resulting in a decline in health expenditure efficiency.

Higher levels of urbanization are associated with higher efficiency scores. This phenomenon may be attributed to two interconnected ways. First, probably owing to the fact that lifestyle changes (e.g., dietary changes) accompanying urbanization contribute to increased prevalence of chronic diseases (e.g., Non-Communicable Diseases, obesity). This epidemiological transition necessitates more attention should be given to the investment and supervision of urban healthcare for the sake of the stable development of the city [33]. Second, a more developed urban transport network also improves the resident’s accessibility in terms of healthcare service availability, which in turn enhances healthcare expenditure efficiency [34]. Moreover, higher urbanization levels may amplify the scale effect of expenditure efficiency, further promoting efficiency gains [35].

This study has several limitations that provide direction for future research. Due to data availability, it is not possible to extend the analysis over a longer period to observe more stable long-term trends. Subsequent research could be conducted by incorporating more up-to-date data to address this limitation. Furthermore, although a random-effects Tobit model accounts for control for unobserved heterogeneity, the potential endogeneity concerns stemming from omitted variable bias, reverse causality, and among others, could not be entirely eliminated. Future research could enhance the robustness of these findings by adopting more advanced econometric techniques. For instance, leveraging quasi-experimental designs (e.g., difference-in-differences analysis in conjunction with regional healthcare policy reforms) could offer deeper insights into the causal determinants of healthcare expenditure efficiency. Besides, integrating healthcare quality indicators could a more holistic perspective on the efficiency of healthcare expenditures. Future studies could further explore a more comprehensive and detailed indicators to better capture the true quality and efficiency of healthcare services. Ultimately, the present study assumes that the 31 DMUs are independent of each other in the healthcare system production process, without considering the impact of geographical location and spatial dependence among provinces on social health expenditure efficiency. Future research could rectify this shortcoming by employing methods such as spatial Durbin models or spatial data envelopment analysis models.

## Conclusion

This study empirically demonstrates a substantial margin for improving the efficiency of province-level social health expenditure by employing the DEA model, Malmquist index model, and Tobit regression model. Enhancing technical changes through more effective allocation and utilization of health expenditures could lead to notable gains in total factor productivity within the healthcare system. Additionally, the findings highlight the importance of urbanization degree and population density as correlates of efficiency, suggesting that policy adjustments may be necessary to better align with actual conditions in different regions.

## Data Availability

Please contact the authors for data requests.

## Abbreviations

TPFCH: Total Factor Productivity Change
EFFCH: Technical Efficiency Progress Change
TECH: Technical Progress Change
PECH: Pure Efficiency Change
SECH: Scale Efficiency Change
DEA: Data Envelopment Analysis
DMU: Decision-making unit
CRS: Constant Returns to Scale
VRS: Variable Returns to Scale
CCR-DEA: Charne, Cooper, Rhodes data envelopment analysis model
BCC-DEA: Banker, Charnes, Cooper data envelopment analysis model
GDP: Gross Domestic Product
